# Frailty Syndrome in Rural Communities: A Narrative Review and Interviews With Rural Individuals

**DOI:** 10.7759/cureus.55088

**Published:** 2024-02-27

**Authors:** Ryuichi Ohta, Toshihiro Yakabe, Chiaki Sano

**Affiliations:** 1 Community Care, Unnan City Hospital, Unnan, JPN; 2 Family Medicine, Unnan City Hospital, Unnan, JPN; 3 Community Medicine Management, Shimane University Faculty of Medicine, Izumo, JPN

**Keywords:** health services accessibility, aging, practice, attitudes, health knowledge, social determinants of health, rural health, frail elderly

## Abstract

Background

With the global increase in aging populations, frailty syndrome, characterized by decreased strength, endurance, and physiological function, has become a critical issue. This study focuses on rural Japanese communities, where the prevalence of frailty syndrome can be notably high due to factors such as multimorbidity, polypharmacy, and a significant population of elderly individuals. This research addresses the gap in understanding frailty’s manifestations and impacts in rural settings, considering unique challenges such as social isolation, limited healthcare access, and the broader social determinants of health.

Methodology

The study employs a narrative review with PubMed and a thematic analysis of semi-structured interviews with 21 elderly community workers in Unnan City. The analysis used the framework of frailty syndrome affected by physiological, social, psychological, and economic factors. The analysis focused on identifying themes related to the social determinants of health affecting frailty and potential solutions.

Results

The following five themes emerged from the analysis: Aging, Rural Contexts, Isolation, Lack of Knowledge of Frailty Syndrome, and Lack of Help-Seeking Behavior for Frailty Syndrome. Four solution-oriented themes were identified, namely, Public Dialogue and Educational Workshops, Frailty Syndrome Health Meetings, Social Engagement Activities, and Political Advocacy for Accessibility to Community Centers. These findings highlight the critical role of community engagement, education, and infrastructure improvements in addressing frailty syndrome in rural areas.

Conclusions

This study underscores the complexity of frailty syndrome in rural Japanese communities, emphasizing the need for targeted interventions that address the unique challenges faced by these populations. By fostering public dialogue, improving healthcare access, and enhancing social support, it is possible to mitigate the impacts of frailty syndrome and improve the quality of life for elderly residents in rural settings. This research contributes to a deeper understanding of frailty in aging societies and the importance of considering social determinants of health in developing effective solutions.

## Introduction

Frailty syndrome, a complex and multidimensional health issue, has become increasingly significant in aging societies globally [1]. This condition, typified by diminished strength, endurance, and physiological function, heightens the susceptibility of individuals to adverse health outcomes. In societies where the population is predominantly older, frailty syndrome is notably pronounced, with 7.4% in over 65-year-old people [2]. This prevalence is further exacerbated by factors such as multimorbidity and polypharmacy, which are common in elderly patients [3]. Multimorbidity negatively impacts physical and psychological conditions, leading to decreased activity in daily life [4]. Persistent in these conditions, older patients are at a heightened risk of developing frailty syndrome.

Frailty syndrome should be considered through understanding the interplay between frailty syndrome and the social determinants of health (SDH) in rural settings. In these communities, the departure of the younger generation in search of employment and more efficient lifestyles has led to an increase in the proportion of older residents [1]. Consequently, these areas are particularly vulnerable to the effects of frailty syndrome. This study explores the physiological, social, psychological, and economic repercussions of frailty syndrome in rural contexts. By focusing on the rural landscape, with its unique challenges and constraints, this study offers a distinct perspective on the issue of frailty in aging societies.

The problem regarding frailty syndrome in rural contexts is twofold: first, there is a lack of comprehensive understanding of how frailty syndrome manifests and impacts individuals in rural settings, especially given the limited access to healthcare and support services in these areas [5]. Second, there is a significant gap in research regarding the perceptions of rural citizens about frailty syndrome and its relation to their SDH [6]. This gap hinders the development of effective strategies and interventions tailored to the needs of these communities.

Given this context, the central research question of this study is “How do rural citizens in aging societies perceive frailty syndrome, and how do these perceptions relate to their social determinants of health (SDH)?” This research aims to elucidate these perceptions through a review of existing literature and interviews with rural citizens in Japan. Focusing on rural Japanese communities, this study aims to contribute to a more nuanced understanding of frailty syndrome in aged societies and highlight these communities’ specific challenges and needs. The findings of this research can inform targeted interventions and policies that address the unique aspects of frailty syndrome in rural settings, ultimately improving the quality of life for elderly populations.

## Materials and methods

A narrative review was performed regarding frailty syndrome and solutions in rural contexts. In addition, to consider the concrete intervention for frailty syndromes in rural Japanese communities, semi-structured interviews were conducted with over 65-year-old citizens with purposive sampling. Based on the narrative review and thematic analysis of the interviews, the concrete implementation of the problem-solving methods in a rural context was considered.

Setting

The interview for this study was conducted in Unnan City, Shimane Prefecture, Japan. Unnan City is in the eastern part of Shimane and borders Hiroshima Prefecture to the south. Its total land area is 553.1 km^2^ and accounts for 8.3% of Shimane, most of which is covered with forests. A survey conducted in 2020 revealed that the total population of Unnan City was 36,007 (17,316 men and 18,691 women), with 40.01% of the population being older than 65 years. Unnan City has six towns (Daito, Kamo, Kisuki, Mitoya, Yoshida, and Kakeya), and each town has multifunctional autonomy and various functions in social issue management. Furthermore, 30 communities have multifunctional autonomies. In traditional protocols, multiple community groups have specific functions (e.g., community organizing, healthcare, and continual education). Thus, each autonomous community organization comprises directors, sub-directors, and clerks active in three main categories, namely, community organizing, healthcare, and social/environmental development. Community organizing refers to citizen empowerment, effective utilization of local resources, and solving community problems through the efforts of local administrative bodies [7].

Participants

Community workers were recruited through purposive sampling from April 2023 to January 2024. We focused on individuals actively engaged in community organizing, healthcare, and social/environmental development across Unnan City’s six towns. Participants were community workers directly involved in one of the three main categories and residing in the target towns. Those not actively engaged in community work or living outside these areas were excluded. The participants were briefed about the study and provided informed consent to participate.

Data collection

For the narrative review, PubMed was used, and the search terms were “(((older) AND (frailty)) AND ((remote) OR (rural))) AND (social determinants of health).” The search clarified the lack of evidence in rural contexts, and three original articles were selected for the review. In the semi-structured interviews, 21 participants were interviewed.

Analysis

RO reviewed the selected articles in the narrative review and constructed several themes regarding SDH affecting older people’s frailty syndrome [8]. The themes were discussed with TY and refined. The study utilized an in-depth deductive thematic analysis to explore themes related to social isolation, health problems, and solution strategies within rural contexts, reflecting on the themes based on the narrative review [9]. Interviews were meticulously recorded, transcribed verbatim, and then analyzed thoroughly. After every three interviews, RO and TY independently read the transcripts to code and identify emerging patterns and themes respecting themes from the narrative review.

RO initially created a preliminary codebook, continually adapting based on the evolving insights from the transcripts. Concurrently, TY independently reviewed the transcripts, contributing to the coding process. The researchers then conducted extensive discussions to compare and contrast their findings, merge codes, and refine themes. This collaborative approach ensured diverse perspectives in data interpretation. The coding and discussion process continued until saturation was reached, with no new themes emerging. At this final stage, CS, a specialist in community care, joined the analysis, adding another level of expertise and validating the themes for their applicability in community care contexts.

After finalizing the themes, the results were translated from Japanese to English. This translation was carefully undertaken to preserve the nuances and context of the original data, enabling accurate communication of the findings to an international audience.

Reflexivity

The research process was enriched by the diverse expertise within the research team, fostering collaborative interactions between researchers and participants. The team comprised RO, a family physician and public health professional with a master’s degree in public health and family medicine; TY, a director of a non-profit organization with extensive experience in rural community support; and CS, a medical educator specializing in community healthcare management. Each member brought unique insights: RO provided practical experience in rural community health, TY offered a grassroots perspective from decades of supporting isolated individuals in communities, and CS contributed an academic and systematic approach to community healthcare management and education. The team engaged in rigorous discussions to ensure an unbiased approach, constantly challenging and refining each other’s ideas. This process involved exploring alternative viewpoints and reflecting on how their professional and personal experiences might influence data analysis. This reflective practice was critical in mitigating potential biases, fostering a more objective and comprehensive understanding of the study’s findings. The team’s engagement with participants was marked by respect and empathy, valuing shared insights and experiences. This participatory approach facilitated a deeper understanding of the subtleties in rural healthcare perceptions and practices, enhancing the study’s overall depth and relevance.

Ethical considerations

The Unnan City Hospital Clinical Ethics Committee approved the study protocol (approval number: 20220012).

## Results

**Figure 1:**
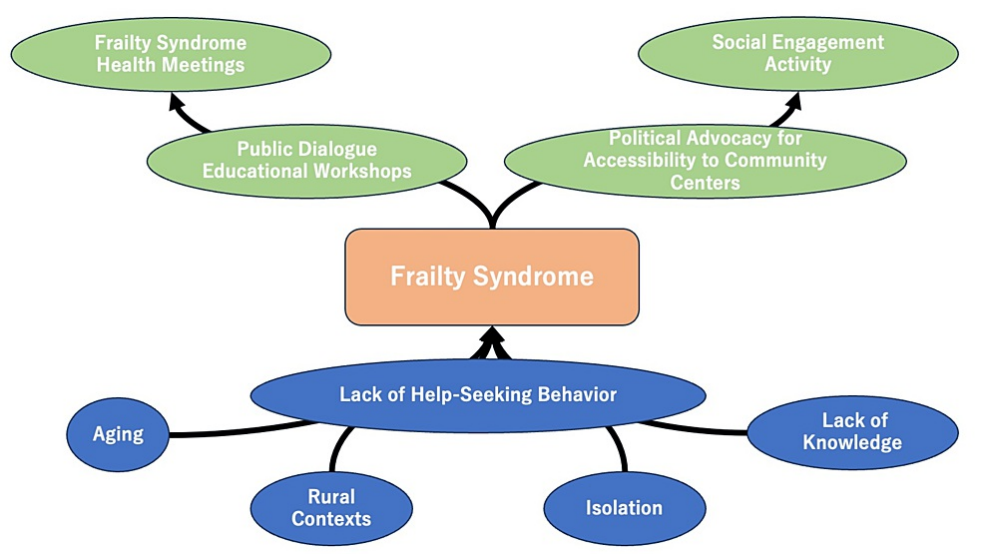
Conceptual figure of the challenges and solutions for frailty syndrome in rural contexts.

The narrative review and thematic analysis developed the following five themes regarding social determinants affecting frailty syndrome in rural communities: Aging, Rural contexts, Isolation, Lack of Knowledge of Frailty Syndrome in Individuals and Communities, and Lack of Help-Seeking Behavior for Frailty Syndrome. Regarding solutions for social determinants affecting frailty syndrome, the following four themes were developed: Public dialogue and educational Workshops, Frailty Syndrome Health meetings, Social Engagement Activities, and Political Advocacy for accessibility to community centers (Figure 1).

Social determinants affecting frailty syndrome in rural communities

Aging

The narrative review shows that aging is one of the critical risks for frailty syndrome [10-12]. As age increases, vulnerability to conditions associated with aging, such as frailty syndrome, becomes more prevalent. Characterized by diminished physiological resilience, frailty syndrome highlights the need for specialized care and attention to cater to the unique needs of our aging global community. One of the participants stated, “I became frail because of aging. In age 60s, I could walk a lot to supermarkets and friends’ houses, but now I cannot go far and bound in my home” (Participant 5, a 70-year-old woman). Rural older people suffer from frailty syndrome because of aging and physiological changes in their bodies.

Rural Contexts

The narrative review shows that living in rural contexts is another critical risk for frailty syndrome [10-12]. The reviewed articles showed that rural settings, with their distinct landscapes and limited infrastructural resources, amplify the hardships experienced by older residents. The isolation inherent to such locales, compounded by the vast distances from urban centers and essential services, exacerbates feelings of neglect and solitude. One of the participants stated, “Living far from the town center, sometimes I feel like the world has forgotten us” (Participant 11, a 75-year-old man). His voice underscores a broader issue: the pressing need to address the unique challenges of aging in rural environments and ensure that every senior feels seen, valued, and connected.

Isolation

Isolation in rural areas is not merely geographical, it is emotional and social. One reviewed article shows that the sparse public transportation coupled with remote healthcare services limits physical mobility and constrains social interactions [12]. Such isolation can amplify feelings of loneliness and alienation among the elderly [12]. One of the participants stated, “Many elderly folks here go days without speaking to anyone. It is heart-wrenching” (Participant 1, a 78-year-old man). His observation emphasizes the profound emotional toll of solitude, underscoring the urgency to address and bridge these gaps in rural communities for the well-being of their aging residents.

Lack of Knowledge of Frailty Syndrome in Individuals and Communities

The previous report shows that frailty syndrome, while prevalent, often remains shrouded in obscurity, leading to missed opportunities for early intervention [12]. It means that individuals might dismiss early symptoms, mistaking them for mere age-related changes. One of the interviewees stated, “Many citizens come to me when their condition has significantly deteriorated. Early detection could have made a world of difference” (Participant 7, a 70-year-old man, physician). The delay in seeking help exacerbates the condition and diminishes the potential for full recovery, emphasizing the imperative for widespread education on the syndrome. Another interviewee stated, “I thought my fatigue and weakness were just a part of getting old. I wish I knew it could be managed” (Participant 9, an 80-year-old woman). Her reflection is a stark reminder that community education is paramount. It is not merely about providing services but ensuring that the community understands, acknowledges, and acts upon health issues such as frailty syndrome.

Lack of Help-Seeking Behavior for Frailty Syndrome

In rural landscapes, the scarcity of robust healthcare and social care systems becomes glaringly evident, and its impact is felt most by those requiring specialized care, such as those with frailty syndrome [12]. The absence of tailored medical facilities and professionals trained in help-seeking behavior directly affects early diagnosis, treatment, and management of the condition. Moreover, the limited social support structures in these regions can exacerbate feelings of isolation among the affected, further hindering their recovery and well-being. In essence, the chasm in healthcare and social behavior provisions in rural areas impedes medical intervention and denies holistic support to those grappling with frailty syndrome. One of the participants stated, “In rural contexts, older people may not know frailty syndrome because of lack of information and communication there. I did not know the reality of the syndrome before listening to medical professionals recently” (Participant 12, a 74-year-old woman).

Solutions for social determinants affecting frailty syndrome

Public Dialogue and Educational Workshops

Empowering a community starts with continual dialogue and education based on the interest. One of the participants stated, “I want to talk with healthcare professionals in communities and find possible ways to prevent frailty in communities” (Participant 2, a 72-year-old woman). Monthly dialogue about health and frailty can be a powerful tool for raising awareness about frailty syndrome. These dialogues about health can provide citizens with accurate, relevant, and context-specific information about their health issues. Family physicians can be essential facilitators of the dialogue and behave citizen-centered for conveying frailty and health information for them to use. Thus, family physicians are vital in health dialogue in preventing community frailty.

Frailty Syndrome Health Meetings

Early detection plays a pivotal role in managing frailty syndrome. Organizing monthly health check-up meetings can proactively identify at-risk individuals and provide them with timely interventions. One of the participants stated, “Older people may not have meetings for their health check except for visiting family physicians. If family physicians collaborate with communities, rural older people can improve their frailty conditions” (Participant 6, an 80-year-old man). Collaboration with rural healthcare facilities and health volunteers ensured such meetings have the resources and expertise to screen and advise participants effectively. The community can gauge the syndrome’s prevalence and the intervention strategies’ efficacy by tracking the number of attendees, cases identified, and referrals made.

Social Engagement Activities

Given the pronounced isolation among the elderly in rural settings, establishing weekly exercise meetings can be instrumental. These gatherings can act as support groups, fostering peer interactions, sharing experiences, and offering emotional solace. One of the participants stated, “Each community should have a place to exercise and communicate with community people. To prevent isolation, each sector should collaborate for it” (Participant 9, an 81-year-old woman). Collaborating with local community centers and social workers can imbue these meetings with structured activities, resources, and counseling. Such sessions can also serve as platforms for health professionals to share updates and information about frailty syndrome. Monitoring these gatherings through qualitative stories of impact can offer insights into their effectiveness in mitigating feelings of loneliness and improving overall well-being.

Political Advocacy for Accessibility to Community Centers

Advocating for better public transport can drastically improve the quality of life for the elderly. By engaging with local politicians and policymakers, the community can emphasize the need for policies prioritizing its aging residents’ health and well-being. One of the participants stated, “Our areas lack useful public transportation systems, and many older people resigned their divers’ license. We cannot visit community centers frequently” (Participant 2, an 87-year-old man). This advocacy is not just about infrastructure and fostering an environment of transportation. Monitoring tools can track policy changes, community support, and infrastructural developments, ensuring that advocacy efforts translate to tangible improvements.

## Discussion

The study’s findings contribute significantly to our understanding of frailty syndrome in rural settings, particularly in aging societies like Japan. The thematic analysis reveals intricate links between SDH and frailty, highlighting critical areas that require attention for better management and prevention of the syndrome in rural communities.

Aging and rural contexts can be amplifiers of frailty syndrome. The themes of aging and rural contexts being critical risk factors for frailty syndrome are consistent with existing literature [13-15]. These findings underscore the necessity of developing strategies sensitive to the unique needs and challenges the elderly face in rural settings [16]. The aging population in these areas is more vulnerable due to the cumulative effects of limited healthcare access and infrastructural resources, accentuating the importance of targeted interventions [17].

Isolation and lack of knowledge may impinge on frailty management as one of the barriers. The study’s identification of isolation and lack of knowledge about frailty syndrome as significant barriers resonates with broader challenges in rural healthcare [18]. These factors not only contribute to the worsening of frailty conditions but also hinder early detection and intervention [19]. Rural older people also tend to endure symptoms and negative changes because of privacy issues [20]. They considered that their negative things should not be known by neighborhood [21]. Addressing these issues requires multifaceted approaches that include community engagement, education, and improved healthcare delivery tailored to the needs of rural elderly populations.

The critical role of help-seeking behavior should be recognized and revised effectively. A crucial finding is rural areas’ lack of help-seeking behavior for frailty syndrome. One factor is that frailty syndrome is a relatively new concept for older people, so they may not recognize the presence of frailty among them [22]. This hesitancy or delay in seeking medical help can cause the deterioration of the condition of frailty due to limited healthcare resources, a lack of awareness, or social factors [23]. Early interventions for frailty can prevent progression [24]. Enhancing help-seeking behavior involves improving healthcare access and increasing awareness about the importance and benefits of early intervention in frailty syndrome.

Solutions for frailty syndrome demand a multidimensional approach. The proposed solutions, including public dialogue, health meetings, social engagement activities, and political advocacy, present a holistic approach to addressing frailty syndrome in rural settings [25,26]. These interventions align with the need for community-centric strategies that address frailty’s health and social aspects [27,28]. Notably, the emphasis on political advocacy for better infrastructure and transportation highlights a crucial aspect often overlooked in healthcare discussions [29,30].

This study, while offering valuable insights into the dynamics of frailty syndrome in rural Japanese communities, is limited by several factors. First, its findings are drawn from a specific geographical and cultural setting, potentially limiting the applicability of its conclusions to other rural settings with different social, economic, and healthcare frameworks. Second, the reliance on qualitative methods, though rich in detail, may not capture the full spectrum of experiences and perceptions among older individuals facing frailty. Additionally, the study’s focus on a singular region and a relatively small sample size might not fully represent the diversity of rural experiences across Japan or other aging societies. Future research could benefit from a broader geographical scope and a mixed-methods approach to enhance the generalizability and depth of understanding of frailty syndrome in varied rural contexts.

## Conclusions

This research provides valuable insights into the multifaceted nature of frailty syndrome in rural settings and proposes comprehensive solutions that address both the health and social dimensions of the syndrome. By focusing on the specific needs and challenges of rural elderly populations, the study contributes to the development of more effective, community-centric strategies for managing and preventing frailty syndrome in aging societies.
